# Preclinical Progress of Subunit and Live Attenuated *Mycobacterium tuberculosis* Vaccines: A Review following the First in Human Efficacy Trial

**DOI:** 10.3390/pharmaceutics12090848

**Published:** 2020-09-06

**Authors:** Jacqueline Watt, Jun Liu

**Affiliations:** Department of Molecular Genetics, University of Toronto, Toronto, ON M5S 1A8, Canada; jacqueline.watt@mail.utoronto.ca

**Keywords:** tuberculosis, Bacille Calmette-Guerin, BCG, recombinant *Mycobacterium bovis* BCG, attenuated *Mycobacterium tuberculosis*, subunit vaccine, viral vector vaccine, vaccines, mycobacteria, *M. tb*, TB, novel TB vaccine, clinical trial, preclinical

## Abstract

Tuberculosis (TB) is the global leading cause of death from an infectious agent with approximately 10 million new cases of TB and 1.45 million deaths in 2018. Bacille Calmette-Guérin (BCG) remains the only approved vaccine for *Mycobacterium tuberculosis* (*M. tb*, causative agent of TB), however clinical studies have shown BCG has variable effectiveness ranging from 0–80% in adults. With 1.7 billion people latently infected, it is becoming clear that vaccine regimens aimed at both post-exposure and pre-exposure to *M. tb* will be crucial to end the TB epidemic. The two main strategies to improve or replace BCG are subunit and live attenuated vaccines. However, following the failure of the MVA85A phase IIb trial in 2013, more varied and innovative approaches are being developed. These include recombinant BCG strains, genetically attenuated *M. tb* and naturally attenuated mycobacteria strains, novel methods of immunogenic antigen discovery including for hypervirulent *M. tb* strains, improved antigen recognition and delivery strategies, and broader selection of viral vectors. This article reviews preclinical vaccine work in the last 5 years with focus on those tested against *M. tb* challenge in relevant animal models.

## 1. Introduction

*Mycobacterium tuberculosis* (*M. tb*), the causative agent of tuberculosis (TB), is the global leading cause of death from an infectious agent and one of the top 10 overall causes of death [1]. There were approximately 10 million new cases of TB and 1.45 million deaths in 2018 [1]. 

Among these deaths, 251,000 were HIV-positive individuals [1]. A growing public health threat concerns the spread of rifampicin-resistant and multidrug-resistant tuberculosis, making up approximately 500,000 of the new TB cases in 2018 [1]. Multidrug-resistant tuberculosis is defined by resistance of *M. tb* infection to both first-line treatments of rifampicin and isoniazid.

Currently, Bacille Calmette-Guérin (BCG) is the only approved vaccine for prevention of TB. It was developed in 1921 from a strain of virulent *M. bovis*, which was attenuated by 230 in vitro passages over 13 years [2]. Starting from 1924, the attenuated strain was distributed around the world and used in humans as a vaccine against TB [2]. BCG is a part of the world health organization (WHO) Expanded Program on Immunization (EPI) and remains the most widely used vaccine in human history [3].

Despite broad coverage of childhood BCG vaccination, clinical studies have shown BCG is mainly effective against meningitis and miliary TB in children, with approximately 80% efficacy [3,4,5]. However, for the more common pulmonary TB disease in adults, it has highly variable efficacy, ranging from 0–80% [3,6]. Evidently, there is a crucial need for an improved vaccine to prevent TB.

Strategies to improve TB vaccines include the development of subunit and live attenuated vaccines. Most new candidates are subunit vaccines for which selected *M. tb* antigens are expressed in replication-deficient viral vectors or are administered as purified protein/adjuvant combinations. A large number of *M. tb* antigens have been tested but none of them have proved to be superior to BCG in animal models [7,8,9,10,11,12]. As a result, subunit vaccines are currently evaluated more as a booster rather than a replacement of BCG [13,14]. MVA85A completed a Phase IIb trial as the first subunit candidate to reach efficacy testing in 2013 [15]. Unfortunately, the results were disappointing as MVA85A was unsuccessful in providing significantly improved protection against TB or *M. tb* infection than in BCG-vaccinated South African infants [15]. This failure has raised questions regarding plausibility of the subunit vaccine approach and further emphasizes the importance of more diversified vaccine research [16]. In the following sections, we will review preclinical progress within the last five years of live attenuated and subunit vaccine strategies as either pre- or post- exposure interventions. Many recent vaccine candidates are being tested in animal models of TB; we will focus on those that examined protective efficacy against *M. tb*.

## 2. Moving Forward from the MVA85A Clinical Trial

Following the results of the MVA85A clinical trial in 2013, there was an increase in effort to develop a new vaccine strategy. As of August 2019, there were 14 new vaccine candidates at various stages of clinical trials [1]. The most advanced being VPM1002, a recombinant BCG vaccine currently in Phase III trials to examine protective efficacy against TB infection [17]. VPM1002 is a urease-deficient recombinant BCG that expresses listeriolysin, a hemolysin produced by *Listeria monocytogenes*, which resulted in leakage of BCG into the cytosol and increased antigen presentation [17]. The safety profile was comparable to BCG, however vaccination of infants with a single dose of VPM1002 induced a similar CD4^+^ T cell response profile to BCG in a Phase II trial in 48 HIV-negative South African newborns [18,19]. An increase in CD8^+^ T cells expressing interleukin (IL)-17 at 6 months postvaccination with VPM1002 was also observed [19].

MTBVAC is the first attenuated vaccine derived from *M. tb* to enter clinical trials. Two genes, *phoP* and *fadD26*, that are associated with cell wall lipid synthesis were deleted in *M. tb* [20]. In 2015, MTBVAC completed a Phase I trial in adults with no history of BCG vaccination and were ESAT-6/CFP-10-seronegative. In this population, vaccination with MTBVAC was shown to have similar safety to BCG [21]. These results are promising for the continued development of attenuated *M. tb* vaccines as they demonstrate that an attenuated *M. tb* vaccine can yield safety similar to BCG. MTBVAC is now in Phase II trials.

## 3. Post-Exposure Vaccines

BCG is given to infants at a single dosage soon after birth. The schedule of BCG vaccination is to minimize the host exposure to *M. tb* and environmental mycobacteria, which would render the effectiveness of BCG [22]. One of the lessons we have learned from the past few years is that developing a better replacement for BCG is a complicated and difficult task. This challenge brought on the idea of post-exposure vaccines as an interim solution. A post-exposure vaccine is designed to be administered to individuals who have been exposed to and are infected with *M. tb* to prevent progression to active TB disease and subsequent *M. tb* transmission. TB is highly transmissible when the disease is in its active form. For the approximately 1.7 billion people latently infected with *M. tb*., there is a 10% chance of progression to active TB in their lifetime [1,23]. This probability increases to 5–10% per year for HIV positive individuals [23]. 

In 2018, two clinical studies showed proof of concept for post-exposure vaccines. M72/AS01 is a combination of a recombinant fusion protein derived from two *M. tb* antigens, PPE18 and PepA, and the AS01 adjuvant system, which is also used in the malaria vaccine and the recombinant zoster vaccine [24]. In a Phase II trial of 3575 QuantiFERON-TB (QFT) positive, HIV-negative adults M72/AS01 prevented the incidence of pulmonary TB disease with 49.7% efficacy after 3 years follow-up [25]. Complementing this study, a Phase II trial including BCG revaccination of 990 QFT-negative adolescents showed a reduced rate of QFT seroconversion with 45.4% efficacy 30 months after revaccination in a high-transmission setting [26]. BCG revaccination showed no significant protection over initial QFT seroconversion. Sustained QFT seroconversion reflects sustained *M. tb* infection [26]. Based on human and animal studies that suggest reversion to a negative tuberculin skin test is associated with a lower risk of TB disease, this study extrapolated that transient QFT seroconversion also represents a lower risk of progression to TB disease [26]. However, they recognized that the clinical significance of QFT reversion remains unclear. Further examination in a prevention of disease study is required to confirm these findings. 

Based on mathematical models of high-incidence countries, a TB vaccine that reduces progression to active TB disease would be most effective for preventing *M. tb* transmission in the short term [27]. However, for optimal control of the TB epidemic, a combination of both a post-exposure and pre-exposure vaccine that prevents initial *M. tb* infection better than BCG remains crucial [27]. 

## 4. Recombinant BCG Vaccines

One theory for the limited efficacy of BCG is due to its inability to elicit a diversified cellular immune response, including a strong CD8^+^ T cell response [28]. This could, in part, be due to BCG’s sequestration in the macrophage phagosome, which limits antigen processing to the major histocompatibility complex (MHC) class II pathway and preferentially activates CD4^+^ T cells [28,29]. BCG also has some major differences from *M. tb*, including the loss of multiple T cell epitopes and the region of difference 1 (RD1) [30,31]. Taking this into consideration, recombinant BCG (rBCG) strains are being developed that include *M. tb* antigens, increase specific immune activation, or are genetically modified to increase antigen presentation. In addition to the recombinant BCG that expresses antigens encoded in RD1 (e.g., ESAT-6 and CFP-10) as discussed in great detail in [23], there have been several new rBCG constructions tested in more recent years that will be discussed here (Table 1).

### 4.1. rBCGs Expressing M. tb Antigens

Rv2212, an adenylyl cyclase that synthesizes cyclic adenosine 3′5′-monophosphate (cAMP) and is absent from BCG, plays an important role in intracellular survival and virulence [32]. rBCG expressing Rv2212 was attenuated in BALB/c mice and improved the percentage of interferon γ (IFNγ)^+^ CD4^+^ and CD8^+^ T cells over the parental BCG strain [32]. However, mice subcutaneously immunized with BCG-Rv2212 and challenged with *M. tb* H37Rv only had significantly reduced lung bacterial loads after 4 months compared to the parental BCG strain (*p* < 0.05), but not after 6 months. There was also no difference between the survival curves of these two groups of animals [32]. Due to the over production of cAMP, BCG-Rv2212 is suggested to partially resemble the actions of a stressed bacilli, similar to the conditions that would be encountered during acute infection [32]. Therefore, this could explain the greater control vaccination with BCG-Rv2212 had at the earlier time point which was not seen at the later time point.

Another strategy to improve the protective efficacy for TB vaccines is to include stage specific antigens. CysVac2 is a fusion protein of acute phase antigen Ag85B and CysD, a highly expressed component of the *M. tb* sulfur assimilation pathway during chronic infection in the mouse lung [37]. When used as an intradermal subunit vaccine, mice immunized with CysVac2 and challenged with *M. tb* H37Rv previously showed slightly reduced bacterial burden than those vaccinated with BCG, although the difference was not statistically significant [37]. Extending from these results, BCG was engineered to express CysVac2 (rBCG:CysVac2). rBCG:CysVac2 elicited improved T helper cell type 1 (Th1) immunogenicity in mice, however protection was similar to the parental BCG strain 4 and 12 weeks after infection with *M. tb* H37Rv [33].

### 4.2. rBCGs Modulating Immunogenicity

There are two main methods that research groups have used to increase BCG immunogenicity: improve antigen presentation via abolishing its ability to hide in the macrophage phagosome or increase the expression of immunogenic proteins. To overcome the limitations with improper phagosome maturation and MHC class II (MHC-II) expression evasion of BCG, a group from Texas generated an autophagy-inducing and toll-like receptor 2 (TLR-2) activating rBCG vaccine by overexpressing Ag85B and the C5 peptide from CFP-10 (BCG^85C5^) [34]. Mice subcutaneously vaccinated with BCG^85C5^ had reduced bacterial loads compared to BCG 30 days after initial challenge (lung *p* = 0.01; spleen *p* = 0.01) and 30 days after re-challenge (lung *p* < 0.05; spleen not significant [n.s.]) with *M. tb* Erdman [34]. This study demonstrated BCG^85C5^’s potential as both a pre- and post-exposure TB vaccine.

PhoP-PhoR is a two-component transcriptional regulator of a number of genes in *M. tb*, including two T cell antigens (Ag85A, PPE18) [35]. A loss of function mutation of *phoP* from BCG-Prague results in lower immunogenicity compared to other BCG strains [35]. BCG-Japan, a strain containing *phoP*-*phoR*, was engineered to overexpress *phoP*-*phoR* and evaluated for its safety, immunogenicity and protection in mice and guinea pigs. rBCG-Japan had comparable safety to the parental strain and was also significantly safer compared to BCG-Pasteur in severe combined immunodeficiency (SCID) mice. rBCG-Japan had an increase in antigen specific IFNγ production and percentage of IFNγ^+^ CD4^+^ T cells compared to the parental BCG strain. Guinea pigs subcutaneously vaccinated with rBCG-Japan had a reduced bacterial load after 8 weeks compared to unvaccinated guinea pigs (lung *p* < 0.05; spleen n.s.) and increased survival by 133% over the parental BCG strain after *M. tb* H37Rv infection [35]. This study suggested that recombinant BCG overexpressing *phoP-phoR* is a promising candidate to replace BCG.

SapM is a secreted acid phosphatase of *M. tb* and has an important role in immunomodulation [36]. Interestingly, recent work on a *sapM* transposon mutant of BCG (*sapM*::Tn BCG) demonstrates better innate control compared to the parental BCG strain, including more rapid recruitment of dendritic cells (DCs) to draining lymphoid organs [36]. The *sapM*::Tn BCG also shows potential as an improved subcutaneous BCG vaccine, increasing long-term survival of *M. tb* H37Rv infected mice [38]. Only a brief investigation of TB-specific IFNγ producing CD4^+^ and CD8^+^ T cells 2 weeks post-vaccination was conducted; further study of the related adaptive immune response to *sapM*::Tn BCG is required [36]. However, this study brings up the question of whether innate immune control may play a larger role than previously considered in protective immunity to TB, a phenomenon that is typically connected to viral infections [39,40].

## 5. Live Attenuated *M. tb* Vaccines

An attenuated live *M. tb*-based vaccine may express a broader antigenic panel and induce immune responses more closely resembling a natural infection of *M. tb* leading to increased long-term protection. However, safety is the main concern with the potential for attenuation reversal. The second Geneva consensus created by the TB community provides guidance for the development of live *M. tb* vaccines, requiring two stable independent mutations [41]. Similar to other vaccine candidates, they must also exhibit comparable safety to BCG in relevant animal models. Recent work has focused on which mutations and which combinations of mutations will not only provide sufficient safety but also be advantageous for inducing a protective immune response (Table 2).

### 5.1. Background M. tb Strain Selection

There are 7 phylogenetic branches of *M. tb*, with lineages 2, 3, and 4 being responsible for the majority of worldwide spread [52]. Determining if there is lineage-dependent protection, moreover selecting which lineage would be the best candidate to develop into a vaccine is an important question when designing a live attenuated *M. tb* vaccine. Most preclinical work has used lineage 4 derived vaccines due to the common lab strain H37Rv being of lineage 4. However, there was little direct evidence to support this selection. 

A recent study generated the well characterized MTBVAC mutations (*phoP* and *fadD26*) in lineage 2 (MTBVAC-2) and 3 (MTBVAC-3) backgrounds and compared their safety and protective efficacy to the original MTBVAC derived from a lineage 4 background [53]. MTBVAC had the best safety profile in a SCID mice survival experiment, showing greater attenuation compared to BCG (*p* < 0.05). MTBVAC-3 had comparable safety to BCG, while MTBVAC-2 was significantly more virulent than BCG (*p* < 0.01) [53]. Mice were then subcutaneously vaccinated with each strain of MTBVAC or BCG and challenged with various *M. tb* strains belonging to all three lineages. Four weeks after challenge with lineage 4 strain H37Rv, all vaccinated mice had similarly reduced bacterial burdens in the lungs compared to unvaccinated mice (*p* < 0.0001) [53]. Against challenge with lineage 2 strain W4-Bejing, MTBVAC and MTBVAC-2 had greater protection than BCG (MTBVAC lung *p* < 0.01; MTBVAC-2 lung *p* < 0.05). Variable protection was shown against challenge with lineage 3 strain HCU3524, with MTBVAC and MTBVAC-3 having the lowest bacterial burdens compared to unvaccinated mice (lung *p* < 0.0001) [53]. 

Overall, the protection afforded by each MTBVAC strain was highly comparable across all *M. tb* challenge experiments, suggesting that the background lineage does not have a large impact on vaccine efficacy. In comparison of MTBVAC-2 and MTBVAC-3, slight lineage-dependent protection was observed against their respective challenge strains. However, MTBVAC was significantly protective in all experiments. With MTBVAC’s lineage independent efficacy and superior safety profile, this study provides the first direct validation for the development of lineage 4 live attenuated vaccines against TB.

### 5.2. Attenuated Vaccines with Multiple Mutations

Live vaccine candidates with multiple attenuating, independent mutations are arguably further along the developmental pipeline, regarding the requirements of the second Geneva consensus [41]. However, a common challenge is optimizing attenuation while maintaining robust protection against TB. One study on a quadruple gene mutant, combining two previously designed mutants of *M. tb*, highlights the importance of this balance [42]. BioA is involved in biotin biosynthesis. A *bioA* mutant of *M. tb* was shown to be highly attenuated, however only provided protection comparable to BCG in guinea pigs [45]. Conversely, a triple gene mutant of *mptpA*, *mptpB*, and *sapM* (*M. tb* Δ*mms*) induced protection in the lungs of guinea pigs but caused pathological damage to the spleen [54]. With the aim to overcome the other’s limitations, a quadruple gene mutant combining these two strategies was designed (*M. tb* Δ*mmsb*) [42]. *M. tb* Δ*mmsb* was highly attenuated in guinea pigs with no recoverable bacilli at 6 weeks and 12 weeks post infection. However, guinea pigs intradermally vaccinated with *M. tb* Δ*mmsb* had significantly higher bacterial burdens than those vaccinated with BCG both 4 weeks (lung *p* < 0.01; spleen *p* < 0.01) and 12 weeks (lung *p* < 0.001; spleen *p* < 0.01) post infection with *M. tb* H37Rv [42]. These findings demonstrate the importance of how *M. tb* is attenuated and its impact on the afforded protection.

*M. tb* Δ*sigE* Δ*fadD26*, an unmarked double mutant, satisfies the requirements of the Geneva consensus for entering human clinical trials [41]. SigE is one of 10 extracytoplasmic function sigma factors and is essential for virulence. *M. tb* Δ*sigE* has previously been shown to be safe and significantly reduce bacterial load in guinea pigs compared to unvaccinated animals (lung *p* < 0.0001; spleen *p* < 0.01), similar to that of BCG following *M. tb* H37Rv infection [46]. *M. tb* Δ*sigE* Δ*fadD26* was significantly more attenuated in SCID mice compared to BCG (*p* < 0.0009) [43]. Subcutaneously vaccinated mice had a reduced bacterial burden compared to BCG up to 4 months post challenge with *M. tb* Harlem genotype 5186 (lung *p* < 0.01.; spleen *p* < 0.01), but its protection against highly virulent *M. tb* Beijing strain K was similar to BCG [43]. Guinea pigs subcutaneously vaccinated with *M. tb* Δs*igE* Δf*adD26* had a similar lung bacterial burden, but a higher spleen bacterial burden (*p* < 0.001) compared to BCG 4 weeks after infection of *M. tb* H37Rv [43].

The only lineage 2-based live attenuated vaccine candidate being developed is derived from the Beijing strain GC1237, which was responsible for outbreaks in the Canary Islands [44]. This vaccine candidate, GC1237 Rv1503c::TnΔ*phoPR*, is inactivated in the *Rv1503c* gene, crucial for surface glycolipid synthesis, and the two-component global regulator *phoP-phoR*, which has established safety in humans in a lineage 4 background [21,44]. Mice subcutaneously vaccinated with GC1237 Rv1503c::TnΔ*phoPR* had similar bacterial loads to those vaccinated with BCG six weeks after challenge with *M. tb* H37Rv. When mice were challenged with lineage 2 strain HN878, only GC1237 Rv1503c::TnΔ*phoPR* vaccinated mice had a significantly reduced bacterial load in the lungs compared to unvaccinated mice 6 weeks post challenge (*p* < 0.05) [44]. This may suggest some lineage-dependent protection, potentially due to variation in antigen expression between *M. tb* Beijing and non-Beijing strains, however further study into this difference in protection is required [44]. Most importantly, they found that GC1237 Rv1503c::TnΔ*phoPR* had comparable virulence to BCG in SCID mice, demonstrating that an attenuated Beijing-based *M. tb* vaccine can be as safe as BCG [44].

### 5.3. Single Mutant Attenuated Vaccines

Single mutant attenuated vaccines require further development to meet safety standards but can indicate which combinations of mutations might be useful. Gene mutations are selected based on their ability to attenuate *M. tb* or for an advantageous phenotype that potentially induces a more robust immune response. For example, lipoproteins are a functionally diverse class of membrane anchored proteins, with deletion mutants shown to be highly attenuated in mice [47]. Once such lipoprotein, LpqS, is highly conserved among pathogenic mycobacteria and when deleted (*M. tb* Δ*lpqS*) is attenuated in guinea pigs. Moreover, when guinea pigs were either subcutaneously or aerosol vaccinated with *M. tb* Δ*lpqS*, bacterial burdens were reduced compared to unvaccinated mice (lung *p* < 0.001; spleen *p* < 0.001) 5 weeks post challenge with *M. tb* H37Rv [47]. The bacterial burden of aerosol vaccinated mice was also significantly reduced in the lungs but not in the spleen compared to subcutaneously vaccinated mice (lung *p* < 0.05) [47]. 

Another study compared two novel vaccine candidates, *M. tb* Δ*mosR* and *M. tb* Δ*echA7* [48]. These genes were selected based on transcriptional studies that revealed their induction following low dose aerosol infection of mice. *mosR* is a transcriptional repressor induced during chronic infection and also shown to be a virulence factor. *echA7* encodes for one of 21 probable enoyl-CoA hydratases annotated in the *M. tb* genome and is induced during the early stages of infection [48]. When mice were subcutaneously vaccinated with either vaccine, both had a reduced bacterial burden in the lungs compared to unvaccinated mice at 30 days (*p* < 0.05) post challenge with *M. tb* Beijing. *M. tb* Δ*mosR* vaccinated mice also had a reduced bacterial load at 60 days (lung *p* < 0.05; spleen *p* < 0.01) [48]. More stringent safety tests are required to evaluate their potential as live vaccine candidates.

Interestingly, a *M. tb* Δ*sigH* mutant was found to be attenuated following aerosol infection of rhesus macaques while having no attenuation phenotype in mice compared to the parental *M. tb* strain [49]. SigH is a key player in the oxidative stress-response pathway of *M. tb* by inducing neutralizing antioxidant production and *M. tb* Δ*sigH* fails to effectively control host oxidants [49]. Macaques aerosol vaccinated with *M. tb* Δ*sigH* had significantly reduced bacterial loads compared to BCG (lung *p* < 0.01) 7 weeks post challenge with *M. tb* CDC1551. Furthermore, >42% of all lung sections from *M. tb* Δ*sigH* vaccinated macaques contained no culturable *M. tb* [49]. *M. tb* Δ*sigH* is a promising live attenuated vaccine candidate, however, additional safety tests are needed as well as determining if further attenuation with a second mutation with alter its protective efficacy.

### 5.4. Naturally Attenuated Mycobacteria Vaccines

The mechanism of BCG’s protective effect to *M. tb* infection could, in part, be due to cross-protection induced by shared antigens. With more than 150 known mycobacteria species, there is potential for another live avirulent strain that can also provide protection to *M. tb* via cross-protection [55]. A selection of avirulent mycobacteria strains: *M. smegmatis*, *M. vaccae*, *M. terrae*, *M. phlei*, *M. triviale*, and *M. tb* H37Ra, were compared to BCG in mice. As expected, different immune responses were induced with the various mycobacterial species [55]. More importantly, subcutaneous vaccination with each strain was also able to control *M. tb* H37Rv infection to different degrees in mice. Four weeks post infection, BCG, *M. smegmatis*, *M. vaccae*, and *M. tb* H37Ra provided the best protection with the lowest lung bacterial loads compared to unvaccinated mice (*p* < 0.01) [55]. In addition, *M. terrae* and *M. triviale* also provided some protection compared to unvaccinated mice (*p* < 0.05) [55]. While the mechanisms of these avirulent strains’ protective immune responses require further study, these results may indicate a greater role for them in developing novel TB vaccines.

Temperature sensitive strains are also ideal for vaccine development as they fail to grow at higher core temperatures of mammals, limiting their ability to infect hosts. Many live viral vaccines have used this property as a mechanism of attenuation [56]. *M. paragordonae* is a naturally temperature sensitive mycobacterial species that is unable to grow in temperatures above 37 °C, human’s core temperature [50]. It was recently shown to exhibit an attenuation phenotype in nude mice compared to BCG 14 days after intravenous infection (lung *p* < 0.001; spleen *p* < 0.001) [50]. *M. paragordonae* also elicited a Th1-skewed immune response, thus was further evaluated for protection against intravenous infection with *M. tb* H37Ra using a subcutaneous homologous prime-boost vaccination strategy. Compared to BCG prime-boost, *M. paragordonae* vaccinated mice had reduced bacterial burdens both 4 weeks (lung *p* < 0.05; spleen *p* < 0.01) and 8 weeks (lung *p* < 0.05; spleen *p* < 0.01) after infection [50]. It is important to note that protection was only evaluated against avirulent *M. tb* and it is unclear whether a protective immune response would also be produced against virulent *M. tb*.

Naturally attenuated mycobacteria have also been genetically modified to enhance their induced immune response. Similar to recombinant BCG strategies, key antigens from *M. tb* can be expressed in avirulent strains with the aim of increasing the protective immune response. One group from Brazil generated a recombinant fusion protein composed of the immunodominant epitopes from Ag85C, MPT-51, and HspX (CMX) and expressed it in *M. smegmatis* mc^2^ 155 (mc^2^-CMX) [51]. They compared the induced immune response of mc^2^-CMX to BCG in the lungs of *M. tb* H37Rv challenged mice and found significant increases in levels of CD4^+^ T cells producing IFNγ (*p* < 0.05) and tumor necrosis factor α (TNFα) (*p* < 0.05) [51]. Production of antibodies specific for CMX in subcutaneously vaccinated mice was also increased (immunoglobulin [Ig] G1 *p* < 0.05; IgG2a *p* < 0.01) [51]. While they showed a reduction in lung injury, further work is needed to evaluate protection efficacy and safety of mc^2^-CMX. 

## 6. Antigen-Adjuvant Vaccines

Adjuvanted vaccines comprise of selected immunogenic antigens from *M. tb* that are combined with an adjuvant system. These vaccines can be targeted to illicit a certain type of immune response based on the adjuvant selected and are generally considered safe and easy to manufacture. However, the major limitation remains the selection and number of *M. tb* antigens included. A single antigen vaccine will most likely not be sufficient to induce long-term protective immunity. Current research has focused on identifying novel antigens as well as designing methods to increase antigen recognition to develop a new generation of multiantigenic subunit vaccines (Table 3).

### 6.1. Novel Antigen Discovery

Antigens are selected based on the levels of their expression in *M. tb* and immunogenicity, measured by an induced IFNγ response. An unbiased immunopeptidomics pipeline for identifying novel antigens presented by MHC was recently developed by a group at Oxford [70]. MHC class I (MHC-I) and MHC-II peptide bound complexes were immunoprecipitated from THP-1 cells infected with BCG and analyzed by mass spectrometry. With this pipeline, they identified 94 peptides presented by MHC-II and 43 peptides presented by MHC-I, from 76 and 41 antigens, respectively. As a proof of concept for this antigen discovery pipeline, three peptides of GlfT2, Fas, and IniB were selected and expressed using two viral vectors (a replication-deficient chimpanzee adenovirus, ChAdOx1, and a replication-deficient modified vaccinia virus Ankara). The resulting viral expression constructs were used as booster vaccines to BCG. When all three peptides were co-expressed by the viral vectors, they conferred significantly greater protection than BCG alone 4 weeks post infection with *M. tb* Erdman (*p* < 0.05) [70]. 

Another group from Oxford selected 15 antigens for validation as a booster vaccine to BCG. These antigens were identified in a human leukocyte antigen (HLA) allele independent genome-wide discovery program using CD8^+^ T cells derived from a diverse population of both active and latent TB patients [71]. The 15 antigens were ranked based on the levels of induced IFNγ secretion by CD4^+^ and CD8^+^ T cells and induced polyfunctional T cells in the lungs and spleen of two mice strains. The top 4 immunogenic antigens (PPE15, PPE51, PE3, and PE12) were expressed by a viral vector (ChAdOx1) and administered intranasally following priming with BCG. They found that when BCG was boosted with PPE15, there was significantly reduced bacterial load 4 weeks post infection with *M. tb* Erdman compared to BCG alone (lung *p* < 0.001; spleen *p* < 0.01) [72]. 

### 6.2. Synthetic Antigen Generation

Synthetic antigen production provides another method to develop novel immunogenic antigens. Traditionally, TB vaccine candidates are based on soluble proteins secreted by *M. tb* that stimulate MHC-restricted Th1-cytokine producing T cells. Synthetic antigens would allow for extension beyond these restrictions and include candidates that would normally be avoided due to insolubility or complex preparations. One such previously unused group of candidate antigens is the mycobacterial membrane protein Large (MmpL) family [57]. A strategy of producing synthetic antigens containing the soluble, extracellular regions of MmpL (SERoM)-1, SERoM-8, and SERoM-10 was recently described and tested as potential vaccine candidates [57]. Dimeric polypeptide proteins containing the two major extracellular loops of each protein were engineered, expressed in, and purified from *Escherichia coli*. Mice were vaccinated subcutaneously with each SERoM or a combination of all 3 and adjuvanted with DDA/MPLA. Four weeks post infection with *M. tb* H37Rv, the bacterial burden in the SERoM-1, 8, 10 combined vaccinated mice was significantly reduced compared to the adjuvant only control (lung *p* < 0.005; spleen *p* < 0.005) [57]. The protection compared to BCG was similar in both the lungs and spleen. 

The cell envelope of *M. tb* contains many complex lipids and glycolipids and their extraction and purification require complicated steps. However, many T cells induced during infection are lipid specific, as such they could be a useful component in subunit vaccines. Diacylated sulfoglycoplipid (AC_2_SGL) was compared to its synthetic analogue (SL37) in combination with phosphatidyl-myo-inositol dimannosides (PIM_2_) and evaluated for protective efficacy in guinea pigs [58]. Guinea pigs were immunized intramuscularly with AC_2_SGL or SL37 combined with PIM_2_ in DDA-TDB. Four weeks post infection with *M. tb* H37Rv, there was a significantly reduced bacterial load in the spleen, but not lungs, compared to unvaccinated mice (spleen *p* ≤ 0.01) [58]. Importantly, they were able to show that natural AC_2_SGL induced comparable protection as SL37. These data provide proof of concept for synthetically generated antigens as potential subunit vaccine candidates. 

### 6.3. Multistage Subunit Vaccines

Expanding the selection of antigens for vaccines to include both replication stage and latency related antigens could be an effective strategy against primary infection and reduce bacterial loads in active and latent TB cases. To generate a broader antigen pool, *M. tb* can be cultured under various metabolic conditions to simulate its different metabolic states during infection. To confirm multistage immunogenicity, recognition of the extracted antigens is tested using T cells from both active and latent TB individuals [73,74]. Recent preclinical and clinical multistage vaccine candidates are well reviewed by Khademi et al. [75]. 

To highlight the potential advantages of a multistage vaccine, one study compared CMFO, a multistage vaccine (Rv2875, Rv3044, Rv2073c, Rv0577), to a single-stage polyprotein CTT3H (CFP-10, TB10.4, TB8.4, Rv3615c, HBHA), and a two-stage polyprotein A1D4 (Rv1813, Rv2660c, Ag85B, Rv2623, HspX) [59]. Mice were subcutaneously vaccinated with each vaccine adjuvanted with DMT or BCG and challenged with *M. tb* H37Rv. Four weeks post infection, CMFO vaccinated mice had significantly reduced bacterial loads, similar to BCG, compared to CTT3H in the lungs (*p* < 0.05) and compared to CTT3H and A1D4 in the spleen (*p* < 0.05) [59]. This study also investigated each stage specific vaccine’s potential to reduce reactivation in a BCG prime-boost model. Mice were primed with BCG then challenged with *M. tb* H37Rv. *M. tb* infection was confirmed by bacterial burden 8 weeks post challenge, then the mice were immunized twice with each vaccine adjuvated with DMT or BCG. Fourteen weeks after vaccination, the bacterial burden was determined and compared to the colony-forming unit (CFU) prior to the boost vaccination. Interestingly, CMFO vaccinated mice had the most significant reduction of bacterial burden compared to BCG (lung *p* < 0.05; spleen *p* < 0.05), with *M. tb* being eliminated in the lung of 3/6 and in the spleen of 5/6 mice [59]. The single stage CTT3H vaccine had the lowest protection across all infection models [59]. These results suggest that a single stage subunit vaccine based only on early secreted antigens is not sufficient to provide protection against primary infection, potentially due to the lack of diversity in presented antigens. Overall, multistage subunit vaccines show promise for developing a new generation of both pre- and post-exposure vaccines against TB.

### 6.4. Protein Antigens against Hypervirulent Strains

BCG is believed to confer poor protection against hypervirulent *M. tb* Beijing strains that are associated with many major TB outbreaks [76,77,78,79,80,81,82]. More of the recent preclinical work is including *M. tb* strains from this family as challenge strains in vaccine protection studies, moving beyond only using *M. tb* H37Rv to improve clinical relevance [60,61]. Using comparative genomics, multiple studies further aimed to determine antigens specific to hypervirulent strains for a booster to BCG [62,63,64,65].

Rv3131, a DosR regulon-encoded putative nitroreductase, was shown to be stably up-regulated during the exponential growth phase and during growth in macrophages with a *M. tb* Korean Beijing (*M. tb* K) isolate [63]. Mice were intramuscularly immunized with Rv3131/GLA-SE and challenged with *M. tb* K. Compared to BCG, it had a similar reduction in bacterial burden 4 weeks post infection, however by 10 weeks post infection, BCG was significantly more protective (lung *p* < 0.001; spleen *p* < 0.05) [63]. MTBK_20640 is a new Beijing-specific proline-glutamic acid (PE) antigen identified and characterized as a dendritic cell activator via TLR-2 mediated pathways and promoted T cell proliferation [62]. Mice intramuscularly immunized with MTBK_20640/glucopyranosyl lipid adjuvant-stable emulsion showed protective potential over the adjuvant only control with a significantly lower bacterial burden (lung *p* < 0.001; spleen *p* < 0.05) 10 weeks following infection with *M. tb* K. However, protection was similar to BCG [62]. When mice were challenged with *M. tb* HN878, there was a similar protective effect as BCG. Overall, lineage 2 specific antigens were effective at inducing antigen specific immunity and gave similar protection to BCG against *M. tb* Beijing strain challenge.

### 6.5. Targeted Recognition Vaccines

In addition to antigen selection, one of the main challenges for novel antigen-adjuvant vaccines is increasing the recognition and presentation of antigens by antigen-presenting cells (APCs) to enhance T cell priming. One of the common strategies to overcome this issue is to select antigens based on their ability to induce a high cellular immune response in TB patients or based on the number of T cell epitopes they contain [66,67]. A more recent strategy has been to directly increase uptake of antigens by APCs with selectively delivering antigens via receptor-mediated recognition [68,69,83,84]. 

Dendritic cells have several types of Fcγ receptors that bind to the Fc domain of IgG molecules [85]. By fusing the Fc-domain of IgG to the selected antigen, antigens can be targeted to Fcγ receptors on APCs, which can increase their uptake and presentation efficiency by approximately 50 to 500-fold [86]. Multiple studies have shown that TB antigens fused to the Fc-domain of mouse IgG improves targeted presentation and increases induced Th1 immunity [68,69,83,84]. A recombinant fusion protein of two highly immunogenic antigens, ESAT-6 and CFP-10, with and without the Fc-domain of mouse IgG2a, was evaluated for immunogenicity in mice following subcutaneous vaccination compared to BCG. The Fc-tagged recombinant protein induced a higher antigen specific Th1 response than BCG (IFNγ *p* < 0.05; IL-12 *p* < 0.05) or ESAT-6:CFP-10 alone (IFNγ *p* < 0.05; IL-12 *p* > 0.05) [69]. This study highlights the advantages of Fc-tagged antigens and their potential to increase the immunogenicity of already highly immunogenic TB antigens. However, no protection studies have been performed to date. Further work is required to determine if this strategy of increasing antigen presentation will lead to increased protection and should be considered for novel antigen-adjuvant vaccines.

## 7. Viral Vectored Vaccines

Different viruses have unique host interactions and resultant induced cellular and humoral immune responses, which can be taken advantage of to generate immunity against other pathogens through the use of attenuated viral vectors [87]. Many viral vectors are in development to express one or multiple *M. tb* antigens (Table 4). The type of virus selected depends on the desired immune response as different viruses elicit various predominant responses, for example, their ability to stimulate CD4^+^ and CD8^+^ T cells, effector T cells, a humoral response, or dendritic cells [87]. However, potential prior immunity to the vector could impact the degree of the induced immune response, which is a critical factor for protection [88]. Relying on selected antigens, viral vectored vaccines also retain the same limitation as antigen-adjuvant vaccines with a lack of diverse *M. tb* antigen presentation.

### 7.1. Modified Vaccinia Virus Ankara and Adenovirus-Based Vectors

Two popular viral vector candidates are modified vaccinia virus Ankara (MVA) and adenovirus (Ad)-based vectors as they are immunogenic, well characterized, and generally considered safe [89,90,91,107]. Despite their promise as TB vaccine vectors, they have yet to be successful in clinical trials. One challenge with MVA is generating enough of an immune response to the antigen within the tolerable dose of the vaccine. A recombinant MVA (rMVA) expressing Ag85B and TB10.4 was developed with the tissue plasminogen activator (tPA) signal sequence to increase antigen expression and secretion [92]. Several DNA vaccines use this method to efficiently drive the expression and secretion of target proteins, however, this is the first MVA vectored vaccine to use the tPA sequence. Comparing the immunogenicity of the subcutaneous rMVA vaccine in mice with and without the tPA signal sequence showed that with the tPA signal, levels of antibodies (Ag85B IgG *p* < 0.05; TB10.4 IgG *p* < 0.05), IFNγ (Ag85B *p* < 0.001; TB10.4 *p* < 0.05), and TNFα (Ag85B *p* < 0.01; TB10.4 *p* < 0.05) were increased [92]. These results suggest that adding a tPA signal in viral vectored vaccines could increase antigen-specific immune responses. Protective efficacy and safety studies are still required.

Another major challenge is overcoming pre-existing immunity to the vector, which occurs with natural exposure to the virus. Natural immunity to Ad vectors is common as exposure to adenovirus occurs frequently among humans. To overcome this issue, chimpanzee adenovirus vectors have been developed [93,94]. A chimpanzee Ad-based vector expressing Ag85A was found to be as safe as a similar human Ad vector after intranasal vaccination while retaining the ability to induce protective immunity against *M. tb* H37Rv in mice previously exposed to human adenovirus (lung *p* < 0.05 compared to human Ad vaccinated mice; lung *p* < 0.01 compared to naïve mice) [94]. Furthermore, to enable more effective boosting with viral vectors, a heterologous prime-boost regimen with MVA and Ad vectors expressing the same *M. tb* antigens has been investigated. Studies suggest that a mixed Ad prime with MVA boost may generate better protective immunity following vaccination with BCG [93,95].

### 7.2. Cytomegalovirus-Based Vaccine

A recent novel cytomegalovirus (CMV)-based vaccine provides proof of concept for complete vaccine mediated immune control [96]. CMV is unique in its ability to induce and maintain lifelong circulating tissue resident effector-differentiated CD4^+^ and CD8^+^ T cells following infection of humans and non-human primates. The rhesus CMV vector (RhCMV) was used to express 9 *M. tb* proteins, 3 from each phase of infection including acute (Ag85A, Ag85B, ESAT-6), latency (Rv1733, Rv3407, Rv2626), and resuscitation (RpfA, RpfC, RpfD) [96]. Rhesus macaques were subcutaneously vaccinated with 2 doses of RhCMV-based vaccine or BCG and challenged with *M. tb* Erdman one-year post vaccination. Vaccination with RhCMV reduced the overall extent of infection by 68% compared to unvaccinated macaques. Overall bacterial burden was also significantly reduced compared to BCG up to 27 weeks post challenge (*p <* 0.05) [96]. Furthermore, 41% showed no TB disease with 10/34 macaques having no culturable *M. tb* from all tissues. This study provides evidence that a long-acting vaccine could control *M. tb* at early stages of infection. It is important to note that there is a major concern of safety regarding CMV-based vaccines and whether an attenuated virus can maintain its ability to elicit robust immunity is still to be determined [108].

### 7.3. Novel Viral Vectors for TB Vaccines in Development

Many other potential viral vectors are being explored as candidates for construction of TB vaccines. With the known shortcomings of MVA and Ad vectors, the goal is to broaden the pool of potential viral vectors in terms of their ability to induce immune responses, natural host immunity, and ability to be genetically modified. A good example of repurposing a well-known vector is lentiviral vector TB vaccines. Lentiviruses are widely used in basic biology and gene therapy due to their efficient gene delivery capabilities [109]. They have also been shown to elicit robust cellular immunity and improve mucosal immunity, making them a potential vector for TB vaccines [97,110]. However, lentivectors retain broad tropism, which could impact their safety. One group generated a novel recombinant lentivirus expressing a fusion antigen of Ag85A-ESAT-6 designed to target dendritic cells [98]. After mice were immunized via footpad injection with one dose of the recombinant lentivector, IFNγ and IL-2 production in spleen lymphocytes was significantly increased compared to the non-DC targeting lentivector (IFNγ *p* < 0.05; IL-2 n.s.) and BCG (IFNγ *p* < 0.001; IL-2 *p* < 0.001) [98]. The dose required to elicit an immune response was lower with the DC targeted lentivector, however more stringent safety tests are required.

A more recent novel vector being developed by a group in Shanghai is a Sendai virus-vectored vaccine expressing Ag85A and Ag85B (SeVAg85AB) [99,100,101]. Sendai virus is a respiratory RNA virus that infects mice and rats. It has low pathogenicity, low levels of pre-existing antibodies, and has been shown to induce DCs maturation, making it an ideal viral vector for a TB vaccine [99]. When mice were primed with BCG and boosted with one intranasal dose of SeVAg85AB, there was a significant decrease in their lung bacterial burdens 5 weeks post infection with *M. tb* H37Rv compared to BCG alone (*p* < 0.05) [100]. 

Additional novel vectors include vesicular stomatitis virus (VSV) and human parainfluenza virus (HPIV). VSV is an easily genetically modifiable RNA virus with the potential to be delivered as a needle free mucosal vaccine [102]. HPIV is a respiratory virus that infects epithelial cells. HPIV type 5 (HPIV-5) has been shown to be safe, inexpensive to produce, and effective as a vaccine against influenza, respiratory syncytial virus, and rabies in animals [103]. In a long-term protection study, mice primed with BCG and boosted with an intranasal dose of HPIV-5 expressing either Ag85A or Ag85B had a significantly reduced lung bacterial burden compared to naïve mice 4 weeks (*p* < 0.01), 9 weeks (*p* < 0.05), and 47 weeks (*p* < 0.05) post infection with *M. tb* Erdman [103]. Another group investigated the potential mechanism of how intranasal immunization with HPIV-2 expressing Ag85B induces a protective immune response in mice [103,104]. They demonstrated that inducible bronchus-associated lymphoid tissue (iBALT) organogenesis plays a role following intranasal vaccination to generate antigen specific T cells and IgA antibodies in the lungs [104]. iBALT organogenesis was independent of Ag85B expression, highlighting the advantage of HPIV-2 as a viral vector. 

### 7.4. Bacterial Vector Vaccines

As an alternative to viral vector vaccines, attenuated bacterial vector vaccines are also being considered. An intriguing bacterial vector for TB vaccine development is attenuated *Listeria* [105,111]. Listeria-vectored vaccines are well established to be safe in immunocompetent humans, inexpensive to produce, present antigens to both MHC-I and MHC-II, and importantly, prior immunity to the vector also does not affect its efficacy unlike viral vectors [105]. Three variously attenuated live recombinant *Listeria monocytogenes* vectors (rLmI, rLmII, rLmIII) all expressing Ag85B were evaluated for protective efficacy as a boost to BCG in mice and guinea pigs [105]. Mice were boosted with 2 intradermal doses of all rLms at 3 and 6 weeks. Compared to BCG alone, rLmIII boosted mice had the longest protective effects with significantly reduced bacterial loads 15 weeks post challenge with *M. tb* Erdman (lung *p* < 0.05; spleen *p* < 0.05) [105]. Moreover, guinea pigs boosted with 2 intradermal doses of rLmI had a significantly reduced bacterial burden 10 weeks following both low dose (lung *p* < 0.01; spleen *p* < 0.0001) and high dose (lung *p* < 0.01; spleen *p* < 0.05) *M. tb* Erdman challenge, compared to naïve guinea pigs [105]. All together, this was the first live attenuated bacterial-vectored vaccine to demonstrate protective efficacy as a booster to BCG. 

Lactic acid bacteria, non-sporulating Gram-positive bacteria, have also been widely used as vaccine vectors due to their known safety and well-established genetic toolkit [106]. Specifically, inactivated *Lactobacillus plantarum* was recently investigated as a booster to BCG due to its immunomodulatory effects and adjuvant properties. *L. plantarum* expressing Ag85B-ESAT-6 fused to a DC targeting peptide on the bacterial surface was the first inactivated *L. plantarum* vectored vaccine to show increased protection in the lungs against *M. tb* H37Rv when used as an intranasal boost to BCG (*p* < 0.05) compared to BCG alone [106]. This observed protection may be due, in part, to high levels of IFNγ, high frequency of polyfunctional T cells, and production of lung IgA [106]. However, further studies are required to determine the best immunogenic design for its use as a candidate TB vaccine [106].

## 8. Conclusions

Tuberculosis remains a significant global health problem and a top priority of WHO to reduce the global incidence and death rates [1]. The current preventive strategy, infant BCG vaccination, is widely used around the world; however, it only provides immunity until 10 to 15 years of age [112,113,114]. A more effective pre- and post-exposure vaccine to BCG would be the single greatest advantage to end the global TB epidemic [115]. However, to achieve this goal, many hurdles remain to be overcome. A major hindrance is the lack of a known immune correlate of protection in animals and humans [116]. As evidenced by many clinical and preclinical studies, robust immunogenicity does not always translate to improved protective efficacy [26,35,102]. Another hurdle is the lack of an animal model that faithfully recapitulates the spectrum of human TB disease including latent TB infection (LTBI) [117]. Interest for developing a controlled human infection model of TB is increasing as a more reliable assessment of vaccines’ efficacy at an early stage of development [116,117,118].

Despite these challenges and the lack of enticing results from clinical trials of TB vaccine, over the past 5 years, there has been accelerated progress in designing and testing new vaccine strategies. A variety of novel antigen discovery and engineering methods, repurposing of strategies effective at combating other pathogens, and optimizing presentation of well characterized immunodominant *M. tb* antigens have all shown potential in preclinical investigations. While not discussed here, the route of vaccination is also being re-evaluated. For example, a recent study found that BCG administered by intravenous route is highly protective in macaques [119]. With more advanced knowledge of *M. tb* biology and TB disease, we remain hopeful that a more effective TB vaccine will eventually be developed. 

## Figures and Tables

**Table 1:** Preclinical recombinant Bacille Calmette-Guérin (BCG) vaccines and their protective efficacy against *M. tb* challenge.

Name	Description	Administration	Challenge	Results of Testing	Reference
rBCGs Expressing *M. tb* Antigens					
BCG-Rv2212	BCG expressing Rv2212, an adenylyl cyclase from *M. tb*	Subcutaneous	*M. tb* H37Rv	Mice: reduced bacterial load after 4 months but not 6 months post infection and no difference in survival curves compared to BCG	[32]
rBCG:CysVac2	Recombinant BCG expressing CysVac2, a fusion protein of Ag85A and CysD	Intradermal	*M. tb* H37Rv	Mice: similar bacterial burdens in the lungs both 4 and 12 weeks post infection compared to BCG	[33]
rBCGs Modulating Immunogenicity					
BCG^85C5^	BCG overexpressing Ag85A and C5 peptide of CFP-10	Subcutaneous	*M. tb* Erdman	Mice: reduced bacterial loads 30 days after initial infection and 30 days after re-infection compared to BCG	[34]
rBCG-Japan/PhoPR	Recombinant BCG overexpressing PhoP-PhoR	Subcutaneous	*M. tb* H37Rv	Guinea pigs: reduced bacterial loads 8 weeks post infection compared to unvaccinated guinea pigs and an increased survival of 133% compared to BCG	[35]
*sapM*::Tn BCG	*sapM* transposon mutant of BCG that abolishes its expression	Subcutaneous	*M. tb* H37Rv	Mice: increased survival compared to BCG	[36]

BCG: Bacille Calmette-Guérin; *M. tb*: *Mycobacterium tuberculosis*.

**Table 2:** Preclinical live attenuated *M. tb* vaccines and their protective efficacy against *M. tb* challenge.

Name	Description	Administration	Challenge	Results of Testing	Reference
Attenuated Vaccines with Multiple Mutations					
*M. tb* Δ*mmsb*	Quadruple *M. tb* H37Rv gene mutant of *mptpA*, *mptpB*, *sapM*, and *bioA*	Intradermal	*M. tb* H37Rv	Guinea pigs: increased bacterial loads at both 4 and 12 weeks post infection compared to BCG	[42]
*M. tb* Δs*igE* Δ*fadD26*	*M. tb* H37Rv double mutant of *fadD26* and *sigE*	Subcutaneous	*M. tb* Harlem genotype 5186, *M. tb* Beijing strain K and *M. tb* H37Rv	Mice: reduced bacterial loads up to 4 months post M. tb Harlem genotype 5186 infection and up to 8 weeks post *M. tb* Beijing strain K compared to BCG Guinea pigs: similar bacterial burden 4 weeks post *M. tb* H37Rv compared to BCG	[43]
GC1237 Rv1503c::TnΔ*phoPR*	*M. tb* GC1237 with inactivated Rv1503c and *phoP-phoR* genes	Subcutaneous	*M. tb* H37Rv and HN878	Mice: similar bacterial loads 6 weeks post *M. tb* H37Rv infection and reduced bacterial loads 6 weeks post infection with HN878 compared to BCG	[44]
Single Mutant Attenuated Vaccines					
*M. tb* Δ*bioA*	*M. tb* H37Rv single mutant of *bioA*	Intradermal	*M. tb* Erdman	Guinea pigs: slightly higher bacterial loads post infection compared to BCG	[45]
ST28/*M. tb* Δ*sigE*	*M. tb* H37Rv single mutant of *sigE*	Intradermal	*M. tb* H37Rv	Guinea pigs: reduced bacterial loads similar to BCG 30 days post infection,	[46]
*M. tb* Δ*lpqS*	*M. tb* H37Rv single mutant of lipoprotein LpqS	Subcutaneous and aerosol	*M. tb* H37Rv	Guinea pigs: reduced bacterial loads 5 weeks post infection via both vaccination routes compared to unvaccinated mice	[47]
*M. tb* Δ*mosR*	*M. tb* CDC1552 single mutant of *mosR*	Subcutaneous	*M. tb* Beijing	Mice: reduced bacterial loads 30 and 60 days post infection compared to unvaccinated mice	[48]
*M. tb* Δ*echA7*	*M. tb* H37Rv single mutant of *echA7*	Subcutaneous	*M. tb* Beijing	Mice: reduced bacterial loads 30 days post infection compared to unvaccinated mice	[48]
*M. tb* Δ*sigH*	*M. tb* CDC1551 single mutant of *sigH*	Aerosol	*M. tb* CDC1551	Rhesus macaques: reduced bacterial loads 7 weeks post infection compared to BCG, with >42% of all lung sections having no culturable *M. tb*	[49]
Naturally Attenuated Mycobacteria Vaccines					
*M. paragordonae*	Naturally temperature sensitive mycobacterium species that only grows below 37 °C	Subcutaneous homologous prime-boost	*M. tb* H37Ra	Mice: reduced bacterial loads both 4 and 8 weeks compared to BCG	[50]
mc^2^-CMX	M. smegmatis mc^2^ 155 expressing epitopes from Ag85C, Mpt-51, and HspX	Subcutaneous	*M. tb* H37Rv	Mice: reduced lung injury similar to BCG 70 days post infection	[51]

BCG: Bacille Calmette-Guérin; *M. tb*: *Mycobacterium tuberculosis*.

**Table 3:** Preclinical antigen-adjuvant vaccines and their protective efficacy against mycobacterium challenge.

Name	Description	Administration	Challenge	Results of Testing	Reference
Synthetic Antigen Generation					
SERoM-1, 8 and 10 adjuvanted with DDA/MPLA	Synthetic antigen containing the soluble, extracellular region of MmpL (SERoM)	Subcutaneous	*M. tb* H37Rv	Mice: reduced bacterial loads when vaccinated with a combination of all three SERoM 4 weeks post infection compared to adjuvant only	[57]
SL37 combined with PIM_2_ adjuvanted with DDA-TDB	Synthetic analogue of diacylated sulfoglycolipd (SL37) with phosphatidyl-myo-inositol (PIM_2_)	Intramuscular	*M. tb* H37Rv	Guinea pigs: reduced bacterial loads 4 weeks post infection compared to unvaccinated mice	[58]
Multistage Subunit Vaccines					
CysVac2 adjuvanted with MPL/DDA	Fusion protein of CysD, the gene encoding the first step of the *M. tb* sulfur assimilation pathway, and Ag85B	Subcutaneous boost to BCG	*M. tb* H37Rv	Mice: similar bacterial burden compared to BCG 4 weeks post infection, when used as a booster vaccine there was no reduction of bacterial loads compared to BCG and adjuvant control	[37]
CMFO adjuvanted with DMT	Multistage vaccine containing Rv2875, Rv3044, Rv2073c, and Rv0577	Subcutaneously	*M. tb* H37Rv	Mice: bacterial loads similar to BCG 4 weeks post infection, but reduced compared to a two-stage and single-stage polyprotein BCG primed *M. tb* infected mice: reduced bacterial loads 14 weeks post boost vaccination compared to BCG, with no culturable *M. tb* in lungs of 3/6 and spleens of 5/6 mice	[59]
Protein Antigens Against Hypervirulent Strains					
Rv2299c-ESAT-6 adjuvanted with DDA	A heat-shock protein 90 family fused with ESAT-6	Subcutaneous boost to BCG	*M. tb* HN878	Mice: reduced bacterial loads 16 weeks post infection compared to BCG alone	[60]
GrpE adjuvanted with IFA	Cofactor of heat-shock protein 70 in the DnaK operon, encoded by rv035	Subcutaneous	*M. tb* Korean Beijing	Mice: reduced bacterial loads similar to BCG both 4 and 8 weeks post infection	[61]
MTBK_20640 adjuvanted with glucopyranosyl lipid	Beijing-specific proline-glutamic acid	Intramuscular	*M. tb* HN878	Mice: reduced bacterial loads 10 weeks post *M. tb* Korean Beijing compared to adjuvant only, and reduced bacterial loads similar to BCG post *M. tb* HN878 infection	[62]
Rv3131 adjuvanted with GLA-SE	DosR regulon-encoded putative nitroreductase, upregulated during macrophage infection of *M. tb* Korean Beijing	Intramuscular	*M. tb* Korean Beijing	Mice: reduced bacterial loads at 4 weeks but not 10 weeks post infection compared to BCG	[63]
InsB adjuvanted with MPL-DDA	An ESAT-6 like antigen belonging to the Mtb9.9 protein subfamily within the Esx family from *M. tb* Korean Beijing strain	Subcutaneous	*M. tb* Korean Beijing	Mice: reduced bacterial loads similar to BCG both 4 and 9 weeks post infection	[64]
PE/PPE peptide ESAT-6 fusion protein	Consensus CD4+ T cell epitopes of PE/PPE proteins fused to ESAT-6	Subcutaneous	*M. tb* HN878	Mice: reduced bacterial loads 15 weeks post infection compared to unvaccinated mice	[65]
Targeted Recognition Vaccines					
ADK adjuvanted with DDA	Adenylate kinase, Rv0733	Subcutaneous	*M. tb* H37Rv	Mice: slightly increased bacterial load compared to BCG 4 weeks post infection, but reduced pathological score	[66]
BM adjuvanted with IFA	Fusion protein of EsxB, EsxD, EsxG, EsxU, and EsxM	Subcutaneous	*M. bovis* BCG	Mice: reduced bacterial loads 3 weeks post infection compared to unvaccinated mice, bacterial loads were also slightly reduced compared to prominent antigens PPE18, EsxB, and Ag85A	[67]
CFP10:Fcγ2 adjuvanted with TDB-DDA	Fusion of the Fc-domain of mouse IgG2a to CFP-10	Subcutaneous	n/a	Mice: increased levels of IFNγ and IL-12 compared to BCG, when used as a booster Th1 response was greater than BCG alone	[68]
Fc-tagged recombinant fusion protein of ESAT-6 and CFP-10	Fusion of the Fc-domain of mouse IgG2a to target antigens to Fcγ receptors on APCs	Subcutaneous	n/a	Mice: greater Th1 type specific immune response compared to BCG	[69]

BCG: Bacille Calmette-Guérin; *M. tb*: *Mycobacterium tuberculosis*; MmpL: mycobacterial membrane protein Large family; IgG2a: immunoglobulin G2a; APCs: antigen presenting cells; n/a: not applicable; IFNγ: interferon γ; IL-12: interleukin-12; Th1: T helper cell type 1.

**Table 4:** Preclinical viral vectored vaccines and their protective efficacy against mycobacterium challenge.

Name	Description	Administration	Challenge	Results of Testing	Reference
Modified Vaccinia Virus Ankara and Adenovirus-Based Vectors					
MVATG18377	MVA-based vaccine expressing 14 *M. tb* antigens as 3 protein fusions: RpfB-RpfD-Ag85B-TB10.4-ESAT-6, SF-Rv2029-Rv2626-Rv1733-Rv0111 and SR-Rv0569- Rv1813-Rv3407-Rv3478-Rv1807-TMR	Subcutaneous and intramuscular	n/a	Mice: induction of CD4^+^ and CD8^+^ T cells targeting a broad spectrum of epitopes following subcutaneous immunization Rhesus macaques: broad and potent cellular immune response following intramuscular immunization	[89]
rMVA.*acr*	Recombinant MVA expressing α-crystallin of *M. tb*	Intradermal, intramuscular, or intranasal boost to BCG	*M. tb* H37Rv	Guinea pigs: intradermal boost had the most reduced bacterial burden 6 weeks post infection compared to BCG alone; intramuscular boost had the next most reduced bacterial burden while intranasal boost had no improvement	[90]
AdHu5Ag85A	Recombinant human serotype 5 Ad expressing Ag85A	Intramuscular, intratracheal or aerosol boost to BCG	*M. tb* Erdman	Rhesus macaques: greatest reduced bacterial burden up to 20 weeks post infection and increased survival following intramuscular boost compared to unvaccinated macaques	[91]
MVATH4	Recombinant MVA expressing Ag85B and TB10.4 with the tissue plasminogen activator signal sequence	Subcutaneous	n/a	Mice: greater Th1 type specific response and antibody response compared to rMVA without the tissue plasminogen activator signal sequence	[92]
ChAdOx1.85A-MVA85A	First immunization with replication-deficient chimpanzee Ad expressing followed by immunization with MVA, both expressing Ag85A	Intranasal ChAdOx1.85A and intranasal/intradermal MVA85A combination boost to BCG	*M. tb* Erdman K01	Mice: similarly reduced bacterial loads 4 weeks post infection compared to BCG alone regardless of vaccination route; better protection than either viral vector boost alone	[93]
AdCh68Ag85A	Chimpanzee Ad 68-based vector expressing Ag85A	Intranasal	*M. tb* H37Rv	Mice: retained protective immunity in mice previously exposed to human adenovirus	[94]
AdH4-MVAH4	Recombinant Ad prime and MVA boost both expressing Ag85B-TB10.4 fusion protein	Subcutaneous heterogenous prime-boost	n/a	Mice: greatest specific Th1 immunity compared to either vector alone or an MVA-Ad prime boost regime	[95]
Novel Viral Vectors for TB Vaccines					
RhCMV	Rhesus cytomegalovirus vector expressing 3 acute phase antigens: Ag85A, Ag85B, ESAT-6, 3 latency antigens: Rv1733, Rv3407, Rv2626, and 3 resuscitation antigens: RpfA, RpfC, RpfD	Subcutaneous	*M. tb* Erdman	Rhesus macaques: reduced bacterial loads up to 27 weeks post infection compared to BCG, overall extent of infection was reduced by 68% with 10/34 macaques having no culturable *M. tb*	[96]
LV vF/85A	Lentivector expressing nuclear factor-B activator vFLIP and Ag85A	Homologous subcutaneous prime and intranasal boost	*M. bovis* BCG	Mice: no reduction in bacterial loads 3 weeks post infection compared to BCG	[97]
LV-AEG/SVGmu	Recombinant lentivirus expressing a fusion antigen of Ag85A-ESAT-6 designed to target dendritic cells	Footpad injection - combination of intradermal and subcutaneous	n/a	Mice: increase IFNγ and IL-2 production in spleen lymphocytes compared to non dendritic cell targeting control	[98]
SeVAg85AB	Sendai virus expressing Ag85A and Ag85B	Intranasal boost to BCG	*M. tb* H37Rv	Mice: reduced bacterial loads 5 weeks post infection compared to BCG alone	[99,100,101]
VSV-846	Vesicular stomatitis virus expressing triple fusion protein TFP846 (Rv3615c-Mtb10.4-Rv2660c)	Intranasal	*M. bovis* BCG	Mice: reduced bacterial loads 24 weeks post infection compared to BCG	[102]
HPIV-5	Human parainfluenza virus type 5 expressing Ag85A or Ag85B	Intranasal boost to BCG	*M. tb* Erdman	Mice: reduced bacterial loads 4 and 9 post infection comped to unvaccinated mice, and slightly better protection than BCG at 47 weeks post infection	[103]
Ag85B-rHPIV2	Human parainfluenza virus type 2 expressing Ag85B	Intranasal	n/a	Mice: organogenesis of inducible bronchus-associated lymphoid tissue is important for induction of antigen specific T cells and IgA antibodies independent of Ag85B expression	[104]
Bacterial Vector Vaccines					
rLmIII	Attenuated live recombinant *Listeria monocytogenes* expressing Ag85B	Intradermal boost to BCG	*M. tb* Erdman	Mice: reduced bacterial loads up to 15 weeks post infection compared to BCG alone Guinea pigs: reduced bacterial loads 10 weeks post infection compared to unvaccinated guinea pigs	[105]
LP_DC	*Lactobacillus plantarum* expressing Ag85B-ESAT-6 fused to a dendritic cell targeting peptide on its bacterial surface	Intranasal boost to BCG	*M. tb* H37Rv	Mice: reduced bacterial loads 4 weeks post infection compared to BCG alone	[106]

BCG: Bacille Calmette-Guérin; *M. tb*: *Mycobacterium tuberculosis*; MVA: modified vaccinia virus Ankara; Ad: adenovirus; n/a: not applicable; IgA: immunoglobulin A; Th1: T helper cell type 1; rMVA: recombinant MVA; IFNγ: interferon γ; IL-2: interleukin-2.
